# Transcriptomics analyses reveal the key genes involved in stamen petaloid formation in *Alcea rosea* L.

**DOI:** 10.1186/s12870-024-05263-6

**Published:** 2024-06-14

**Authors:** Yuanzhi Luo, Yifeng Li, Xiancai Yin, Wanqing Deng, Jianwei Liao, Yuanzhi Pan, Beibei Jiang, Hongchen Yang, Keying Ding, Yin Jia

**Affiliations:** College of Landscape Architecture, Sichuan Agricultural University, Chengdu, 611130 China

**Keywords:** *Alcea rosea* L., Flower morphology, Stamen petaloid, Transcriptomics, Plant hormone

## Abstract

*Alcea rosea* L. is a traditional flower with a long cultivation history. It is extensively cultivated in China and is widely planted in green belt parks or used as cut flowers and potted ornamental because of its rich colors and flower shapes. Double-petal *A. rosea* flowers have a higher aesthetic value compared to single-petal flowers, a phenomenon determined by stamen petaloid. However, the underlying molecular mechanism of this phenomenon is still very unclear. In this study, an RNA-based comparative transcriptomic analysis was performed between the normal petal and stamen petaloid petal of *A. rosea*. A total of 3,212 differential expressed genes (DEGs), including 2,620 up-regulated DEGs and 592 down-regulated DEGs, were identified from 206,188 unigenes. Numerous DEGs associated with stamen petaloid were identified through GO and KEGG enrichment analysis. Notably, there were 63 DEGs involved in the plant hormone synthesis and signal transduction, including auxin, cytokinin, gibberellin, abscisic acid, ethylene, brassinosteroid, jasmonic acid, and salicylic acid signaling pathway and 56 key transcription factors (TFs), such as MADS-box, bHLH, GRAS, and HSF. The identification of these DEGs provides an important clue for studying the regulation pathway and mechanism of stamen petaloid formation in *A. rosea* and provides valuable information for molecular plant breeding.

**Supplementary Information:**

The online version contains supplementary material available at 10.1186/s12870-024-05263-6.

## Introduction

The characteristics of petals are of huge significance in ornamental plants. Based on the number of petals, flowers can be divided into single-petal, double-petal, and multi-petal flowers [1]. A flower usually consists of four parts: sepals, petals, stamens, and pistils. Single-petal flowers have only one layer of petals, and the petals are wide and flat. Double-petal and multi-petal flowers have two or more layers of petals. Of note, the stamens or pistils of double-petal flowers mutate into abnormal petals, while the stamens and pistils of multi-petal flowers develop normally [1]. Numerous studies postulate that pistil and stamen petaloid are the most common way to form double-petaled flowers, and the degree of petaloid determines the appearance of flowers with different morphology. Many plants, such as *Eriobotrya japonica*, *Hippeastrum hybridum*, *Lagerstroemia speciosa*, and *Prunus mume* produce a wide variety of flower morphologies because of stamen petaloid organs [2–5]. A double petal is one of the main ornamental traits of flowering organs in angiosperms. Double-petal phenotypes are thus selected by breeders in many species because of the high ornamental value of double-petal flowers [6].

On one hand, the development of plant floral organs is regulated by various genes. In particular, *MADS-box* is an important transcription factor (TF) family that plays an important role in plant growth and development, regulation, and signal transduction [7]. Coen et al. (1991) divided the *MADS-box* TFs controlling flower development into three categories and established the ABC model of flower development, which is regulated by various members of the *MADS-box* family [8]. Class-A genes, *APETALA1* (*AP1*) and *APETALA2* (*AP2*) control the development of sepals. Class-A (*AP1* and *AP2*) and class-B genes, *APETALA3* (*AP3*) and *PISTILLATA* (*PI*), control the development of petals. Class-B (*AP3* and *PI*) and class-C *AGAMOUS* (*AG*) genes control the development of stamens. Class-C (*AG*) genes control the development of pistils [9–13]. Class-D genes, *SEEDSTICK* (*STK*), and class-E genes, *SEPALLATA* (*SEP*), have since been discovered [14, 15]. The ABC model has thus been further improved into the ABCDE model [16]. Class A, B, C, and E genes form a complex called the “tetramer model” [17]. Class-A genes (*AP1* and *AP2*) and class-E genes (*SEP*) control the development of sepals. Class-A (*AP1* and *AP2*), class-B (*AP3* and *PI*), and class-E genes (*SEP*) control the development of petals, while class-C (*AG*) and class-E (*SEP*) genes control the development of pistils. However, class-D genes (*STK*) determine the development of the ovules, and ovules develop into seeds after pollination and have little effect on flower shape [18]. Therefore, this model is also referred to as the ABCE model [19, 20]. The loss of function of ABCE genes in floral organs can lead to homeotic conversion, that is, the transformation of one organ form into another, resulting in developmental changes in the flower organ [21]. Many studies have shown that deletions or mutations of two types of class-B and -C genes are important factors in morphological variation [22–25]. For example, in Ranunculaceae, reduction or elimination of *AP3-3* gene expression is closely related to petal loss [22]. *LMADS1* in *Lilium longiflorum* has high sequence homology with other members of the AP3 family, and ectopic expression of *LMADS1* cDNA truncated with the *MADS-box* domain in transgenic *Arabidopsis thaliana* generated an ap3-like dominant negative mutation, which caused the petals to be converted into sepal-like structures and the stamens to be converted into carpel-like structures [23]. In *A. thaliana*, petals and stamens are absent when the *AP3* and *PI* genes are disrupted by mutation, but under less disruptive *AP3* and *PI* mutations, they exhibit partial conversion of petals to sepals and stamens to carpels [24]. Moreover, *AP3* and *PI* genes directly restrict *AP1* expression early in flower development [25]. Deletion of *AG* results in the transformation of stamens to petals [26–28]. In addition to the *MADS-box* family, a number of TFs have been hypothesized to be potentially involved in petal development. Recently, several RNA-Seq-based studies were performed to characterize the key genes controlling petal development in some non-model plant species, e.g. Lin et al. (2018) selected numerous genes involved in hormone signal transduction pathways and transcription factors as candidate genes for the formation of *N. nucifera* stamen petals through comparative transcriptome sequencing [29]. Fan et al. (2021) performed transcriptomic analysis of single-petal and double-petal *Paeonia lactiflora* flowers and screened out 18 candidate genes involved in the formation and development of petaloid stamens [30]. The study further proposed a hypothetical model of gene expression network regulating the development of petaloid stamens. In a follow-up study, wild-type, “semi-double,” “peony-double”, and “rose double” types of *Camellia sasanqua* were used as the experimental materials and the key pathways and genes associated with double flower patterns regulation were identified by pairwise comparisons, further verifying the ABCE model [31].

Whereas on the other hand, phytohormones can affect the growth and development of floral organs by regulating the expression of genes in their signal transduction and synthesis pathways [32–34]. For example, auxin regulates the expression of *AUX/IAA*, *ARF*, and *GH3*, thereby affecting cell expansion and division, cell elongation and differentiation, and various physiological responses [32]. Cytokinins regulate the expression of *CRE1*, *AHP*, and *B-ARR* to promote cell division and differentiation [33]. In addition to regulating their own signaling pathways, plant hormones may also regulate the expression of other genes related to flower development. Analysis of the *cis*-acting elements in the promoter region of *MADS-box* genes in *Prunus campanulata* ‘Plena’ showed that auxin, abscisic acid, gibberellin, methyl jasmonate, and salicylic acid regulate the transcriptional expression of *MADS-box* genes, which in turn affects floral development and differentiation [34]. Currently, most published studies related to plant hormones have focused on seed germination and dormancy, flower bud differentiation, and floral regulation [35, 36], but the hormone signaling pathways associated with stamen petaloid development still need to be explored in depth.

*Alcea rosea* L., also known as Hollyhock, is a biennial erect ornamental plant of the *Alcea* genus and a member of the Malvaceae family [37]. Most varieties exhibit a tall plant type, usually up to 2–3 m in height, but there are some dwarf varieties. It is native to Sichuan Province, China and usually grows in warm temperate and tropical regions, especially sunny places. It has a strong ability to withstand cold and drought and salt and alkali resistance. Notably, it can grow in soil with a salt content of 0.6% but does not do well in water-logged conditions [38]. *A. rosea* is widely grown worldwide because of its tenacious vitality and rough cultivation management. *A. rosea* is a common ornamental plant with a high aesthetic value because of the rich color and shape of its flower [39–41]. Based on the number of petals, *A. rosea* can be categorized into single-petal and double-petal flowers. The ordinary single-petal flower has only five petals, but the double-petal flower has more than ten petals or even dozens of pieces. The double-petal flowers of *A. rosea* develop from stamen and pistil petaloid. Notably, the stamen petaloid is the most common, and different degrees of stamen and pistil petaloid form different double-petal flower types [42]. Gao et al. (2022) used RNA-seq technology to perform a comparative transcriptomic analysis of the multi-petal red flower (mr) and single-petal red flower (sr) of *A. rosea*. A series of differential expression genes (DEGs) involved in plant hormone synthesis and some key transcription factors (TFs), which are closely associated with the stamen petaloid of *A. rosea*, were identified [43]. However, this previous study authors used whole single-petal and double-petal flowers as sequencing samples, meaning the sample materials contained multiple floral organs that are not associated with the stamen mutation, such as sepals and pistils, which may cause redundant interference in the experimental results [43]. The petaloid characteristics of *A. rosea* should thus be further explored to improve its aesthetic value.

In this study, a RNA-seq-based comparative transcriptomic analysis between stamen petaloid petals and normal petals of double-petal pink flowers of *A. rosea* was conducted to elucidate the molecular mechanism of stamen petaloid development. This study is aimed to investigate whether stamen petaloid development in *A. rosea* is regulated by class-B and -C genes in the ABCE model, to identify key genes associated with stamen petaloid and, to analyze the corresponding regulatory pathways. The findings of this study may contribute to the development of novel varieties, by adding the ornamental value of double-petal flower types, enhancing innovation in the horticulture industry.

## Materials and methods

### Plant materials

**Fig. 1:**
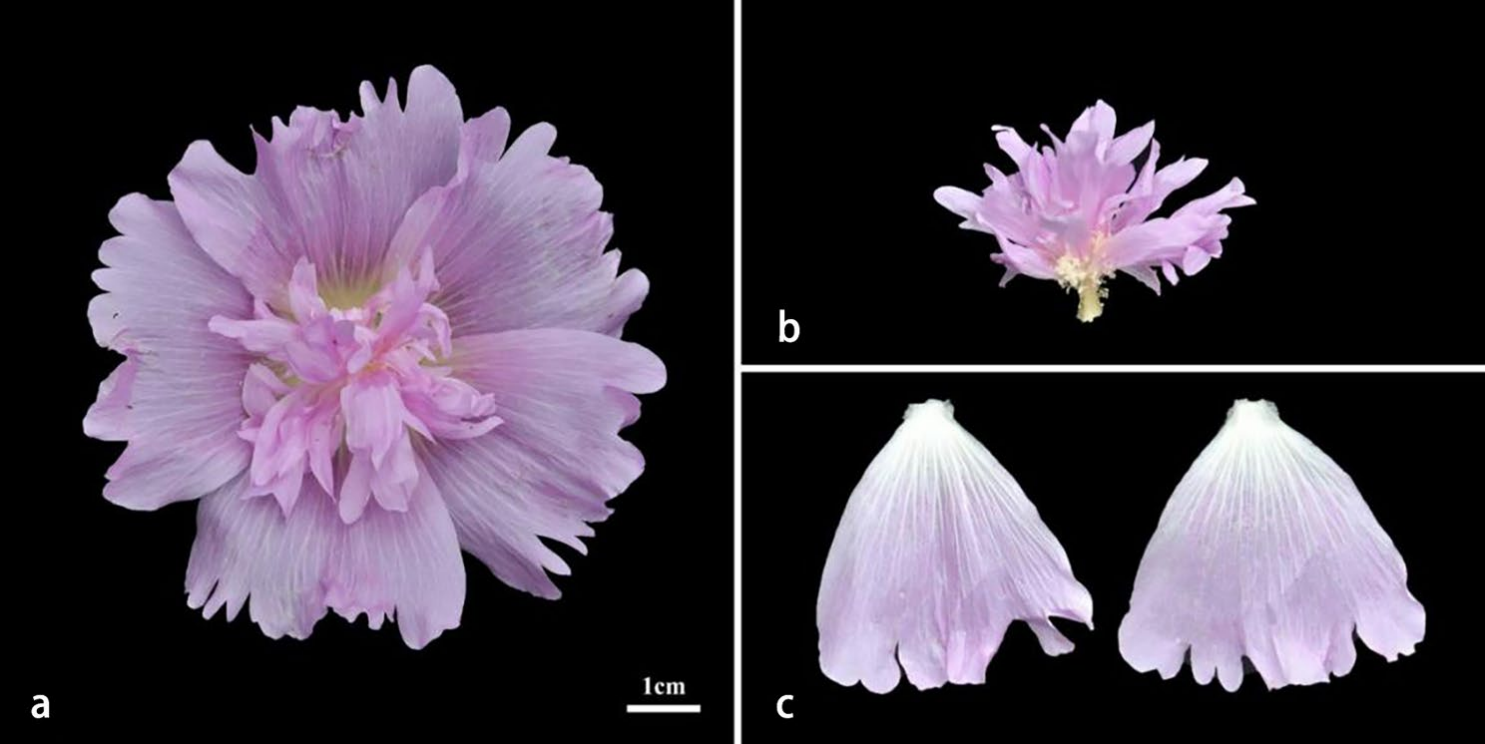
Torch-type flower and petal of *A. rosea.* (**a**) Torch-type flower of *A. rosea*. (**b**) Stamen petaloid petals (PP). (**c**) Normal petals (NP)

The pink *A. rosea* (Fig. 1a) used in this study was cultivated in the Botanical Garden of Chengdu (104°13’ E, 30°76’ N), Sichuan Province, China. When the flowers were in full bloom, the stamen petaloid organs (PP) (Fig. 1b) and normal petals (NP) (Fig. 1c) of the same pink *A. rosea* were sampled, each with three independent biological replicates. All the samples were collected between 8 and 10 AM, frozen in liquid nitrogen, and stored at -80℃ until RNA extraction. The stamen petaloid petals and normal petals were used to construct six libraries for RNA-seq. The six were named NP1, NP2, and NP3 for NP and PP1, PP2, and PP3 for PP.

### RNA extraction, library construction, sequencing, and assembly

Total RNA was extracted from all samples using the RNA Easy Fast kit (DP452) (Tiangen Biochemical Company) following the manufacturer’s instructions. RNA integrity was assessed by the use of 1% gel electrophoresis, a Qubit® 2.0 Fluorometer (Life Technologies, Carlsbad, CA, USA), BioAnalyzer, and a Agilent 2100 RNA Nano 6000 Assay Kit (Agilent Technologies, Santa Clara, CA, USA). The cDNA libraries were prepared using the cDNA Synthesis Kit following the manufacturer’s protocol (Illumina, San Diego, CA, USA). The resulting six cDNA libraries were pair-end sequenced on an Illumina NovaSeq 6000 platform (Illumina), which yielded 150 bp double-ended sequencing reads. The Trimmomatic software was used to remove the duplicate sequences contained in raw reads and bases with a Phred score of less than 20 [44], and by filtering reads by length (> 50 bp) or with only one end [45]. The quality of the original sequencing data of each sample was evaluated based on the raw reads, bases, GC %, Q20, Q30, and average quality. The raw reads containing adapter sequences, low-quality reads (Q value < 20), high N rate (≥ 10%) sequence, and the small fragments of less than 25 bp in length after the adapter and mass trimming were discarded; the remaining reads were defined as clean reads. Finally, the remaining clean reads were assembled into complete unigenes with Trinity (version 2.0.6) following the default parameters for de novo assembly [44].

### Functional annotation of unigenes

All nucleotide sequences of the unigenes were aligned against various databases, including the NCBI non-redundant protein (NR), the NCBI nucleotide (NT), gene ontology (GO), Kyoto Encyclopedia of Genes and Genomes (KEGG), Universal Protein (Uni-prot), and the transcription factor database (TF). The transcript sequences were compared with the NT database using blastn, and the sequences translated into proteins were compared with other databases using diamond, as a way to obtain comprehensive biological information about each individual gene [46].

### Differential expression analysis of unigenes

The expression levels of the unigenes in the RNA-seq data were assessed according to their fragments per kilobase of exon model per million mapped reads (FPKM) values, i.e., the expression level of each unigene was normalized to the number of transcripts per thousand base pairs, after which the resulting *P*-value was used to analyze the difference in expression of individual unigenes in the two groups of samples. The Benjamini-Hochberg method was used to adjust the significant *P*-values obtained in the original hypothesis test, and the *P*-values were adjusted according to their false discovery rate (FDR) test for transcripts that met the following criteria: FDR < 0.05 and fold change (FC) ≥ 2. Finally, unigenes with P-values < 0.05 were identified as differentially expressed genes (DEGs). GO enrichment analysis of DEGs was performed by topGO software [47], i.e., sets of genes with similar GO functions were enriched together by a statistical test algorithm, thus facilitating the study of genes with a particular type of GO function. A GO enrichment scatter map was plotted using the hypergeometric test. KEGG pathways significantly enriched in the DEGs were identified and analyzed using the hypergeometric test [48].

### Validation of RNA-seq data by qRT-PCR

Eleven DEGs were selected for qRT-PCR to verify the reliability of RNA-seq. As reference gene we used the 18 S rRNA gene, previously used in *A. rosea* [43]. The primers for these genes were designed using Primer Premier 5.0 software and are listed in Supplementary Materials 1. The qRT-PCR assay was performed with the Analytik Jena qTOWER 2.2 fluorescence meter (CFXCONNECT, Bio-Rad, Hercules, CA, USA). The qRT-PCR program included pre-denaturation at 95℃ for 5 min, followed by 40 cycles of denaturation at 95℃ for 10 s and annealing at 60℃ for 30 s. The relative expression levels of these genes were calculated using the 2^−ΔΔCt^ method [49]. Each qRT-PCR reaction was performed in triplicate, followed by an analysis of the mean differences.

## Results

### Stamen Petaloid phenotype of *A. Rosea*

Flower shape is one of the most important factors determining the ornamental value of *A. rosea*. The pink double-petal flowers had 20–25 petals, including the outer normal petals and the inner petaloid organs. Stamen column petaloid organs occurred distally, with the lower floral whorl normally developed, and the whole stamen column and stamen petaloid petals were of the torch-like type. Stamen petaloid organs were small and curled, and the outer normal petals were wide and flat; this feature makes floral appearance more attractive and provides a good material for the study of stamen petaloid development in *A. rosea*.

### Quality analysis sequencing data and assembly results

Despite the economic importance of *A. rosea*, no genomic resources are available for this species. As such, the assembly of a de novo transcriptome was necessary to study the molecular mechanisms of stamen petaloid development. In this study, the RNA of NP and PP were sequenced without parameters. A total of 40.28 Gb clean data were generated from the six libraries after filtering and quality testing, the total raw and clean reads, clean data and other parameters of library quality are summarized in Table 1. The Trinity assembly generated 206,188 unigenes of over 200 bp in length with an N50 length of 1208 bp. Additionally, the assembly generated numerous larger unigenes, 102,793 with a length of 200–500 bp, 50,165 with a length of 501–1000 bp, 38,194 with a length of 1001–2000 bp, and 15,036 unigenes longer than 2000 bp.

**Table 1:** Statistics of sequencing data quality evaluation

Sample	NP1	NP2	NP3	PP1	PP2	PP3	Average	Sum
Raw Reads	43,747,940	46,283,838	38,178,658	44,568,776	46,601,862	49,125,592	44,751,111	268,506,666
Clean reads	41,733,014	44,324,672	36,556,328	42,792,300	44,686,676	47,266,718	42,893,285	257,359,708
Bases number (Gb)	6.049	6.455	5.301	6.252	6.518	6.881	6.243	37.456
GC(%)	44.545	44.205	43.850	43.090	43.130	45.360	44.030	
Q20(%)	98.655	98.750	98.645	98.865	98.810	98.865	98.765	
Q30(%)	95.030	95.370	94.985	95.775	95.570	95.810	95.423	
Average quality	33.650	33.725	33.665	33.810	33.765	33.815		
Total clean data (Gb)								40.28
Total numbeer of unigenes (bp)								206,188
N50 length (bp)								1,208

### Annotation of unigenes

**Fig. 2:**
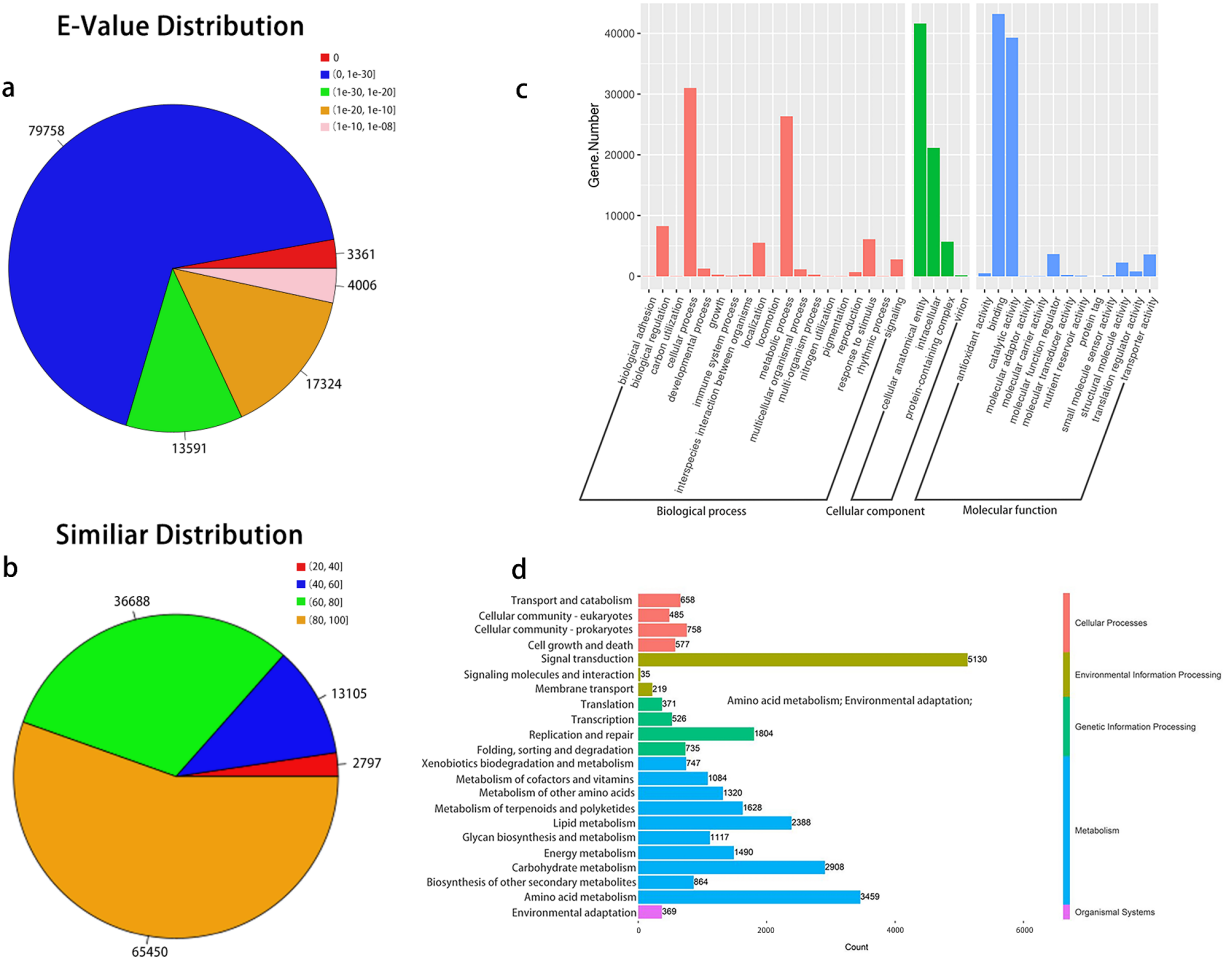
Annotations of unigenes. (**a**) Statistics of e-value distribution in the NR database. (**b**) Statistics of similarity distribution in the NR database. (**c**) GO functional annotation. (**d**) KEGG classification

The functional annotation of the unigenes was achieved with all 206,188 unigenes, against six unigene databases. The NT database annotated the functions of 140,547 unigenes (68.16%), KEGG annotated for 122,304 unigenes (59.32%), Uni-prot annotated for 118,304 unigenes (57.38%), NR annotated for 118,040 unigenes (57.25%), and TF annotated for 43,621 unigenes (21.16%). A BLAST search against the NR database showed that there were 79,758 (67.57%) annotated unigenes with an e-value less than 1e-30 (Fig. 2a), amongst which 65,450 (55.45%) unigenes shared more than 80% similarity with those in the NR database (Fig. 2b). The similarity of the gene sequences of *A. rosea* and closely related species was obtained through comparisons in the NR database. A total of 592 species were compared in this experiment, and the results indicated that species in the family Malvaceae, such as *Gossypium raimondii*, *Gossypium arboreum*, *Gossypium hirsutum*, *Theobroma cacao*, *Herrania umbratica*, *Corchorus capsularis*, and *Corchorus olitorius*, are closely related to *A. rosea* (Supplementary Materials 2).

The genes obtained from the transcriptome database were classified based on their GO and KEGG functions. The GO functions included the cellular component, biological process, and molecular function and were enriched in 84,096 genes (40.7%), 68,547 genes (33.2%) and 93,650 genes (45.4%), respectively (Fig. 2c). KEGG functions included the cell process, environmental information processing, gene information processing, metabolism, and organic system, accounting for 8.64%, 18.78%, 11.98%, 59.31%, and 1.29% of the genes, respectively (Fig. 2d).

### Differential expression analysis of unigenes

**Fig. 3:**
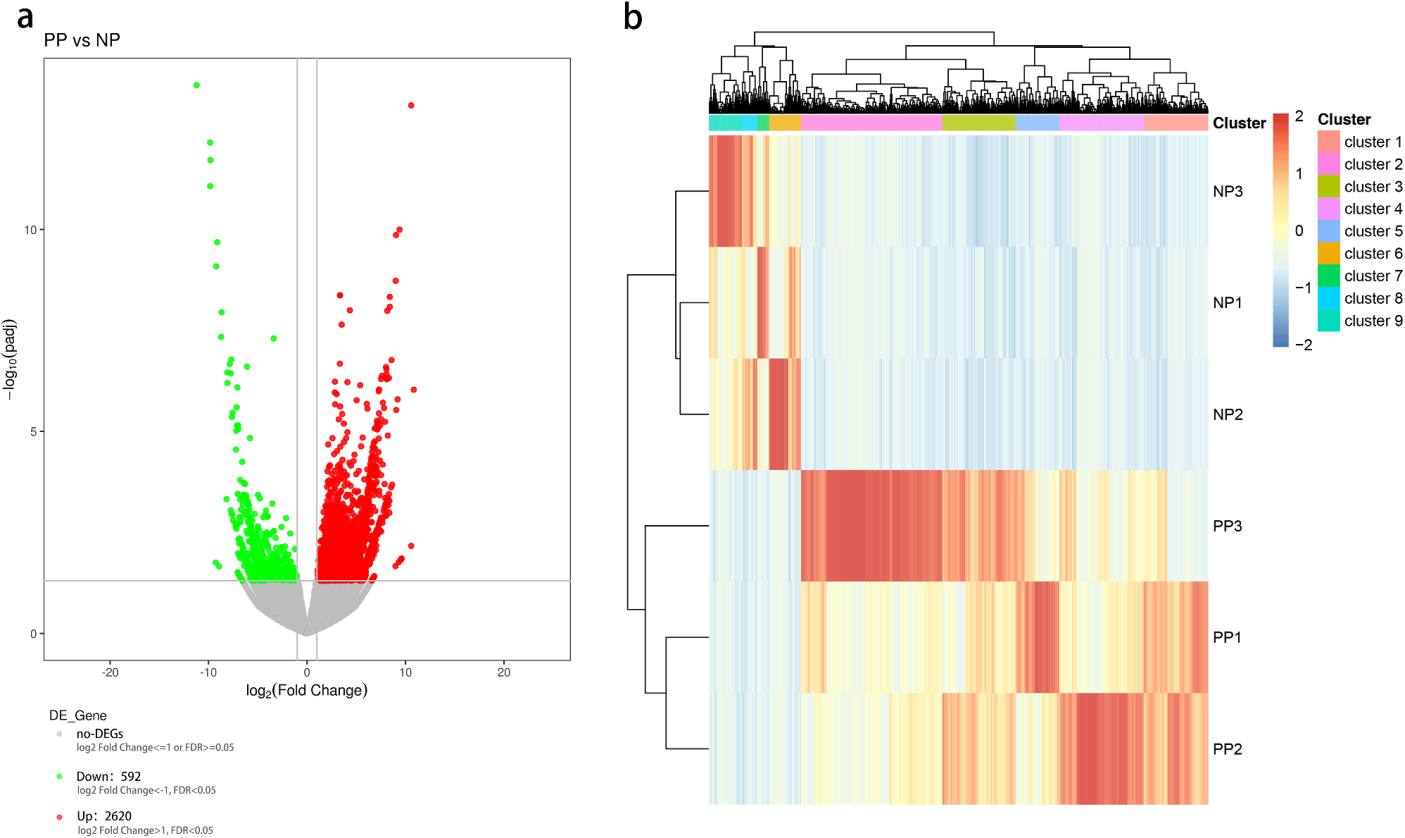
Gene expression comparisons. (**a**) Volcano map of DEGs. The horizontal ordinate denotes the multiple change value of unigenes’ expression difference in PP vs. NP; The vertical ordinate denotes the statistical test value of unigenes’ expression difference in PP vs. NP, that is, the p-value. The dots represent the unigenes: the red dots indicate up-regulation, the green dots indicate down-regulation, and the gray dots indicate no difference. (**b**) Clustering heat map of DEGs.The top is the tree diagram of unigenes clustering. The shorter the distance between the two unigene branches, the closer the expression level. On the left is the tree diagram of sample clustering, and on the right is the name of the sample

To identify the genes associated with the stamen petaloid organs, we conducted a pairwise comparison among petals (NP libraries) and stamen petaloids petals (PP libraries). The FPKM value was used to estimate the level of gene expression. In total, 3,212 DEGs were obtained; 2,620 DEGs were up-regulated, while 592 DEGs were down-regulated (Fig. 3a). The log_10_(reads per kilobase per million mapped reads [RPKM]) values of the unigenes were used for gene expression normalization and hierarchical clustering analysis. In Fig. 3b, genes shown with different colors represent different clusters, and the DEGs were categorized into nine groups/clusters. Genes in the same cluster had similar expression patterns and potentially had similar functions or participated in the same biological process. Among the clusters, five were up-regulated, and the other four were down-regulated in PP vs. NP (Fig. 3b), which may indicate that these DEGs are involved in the stamen-to-petal developmental change through up- or down-regulation, respectively.

**Fig. 4:**
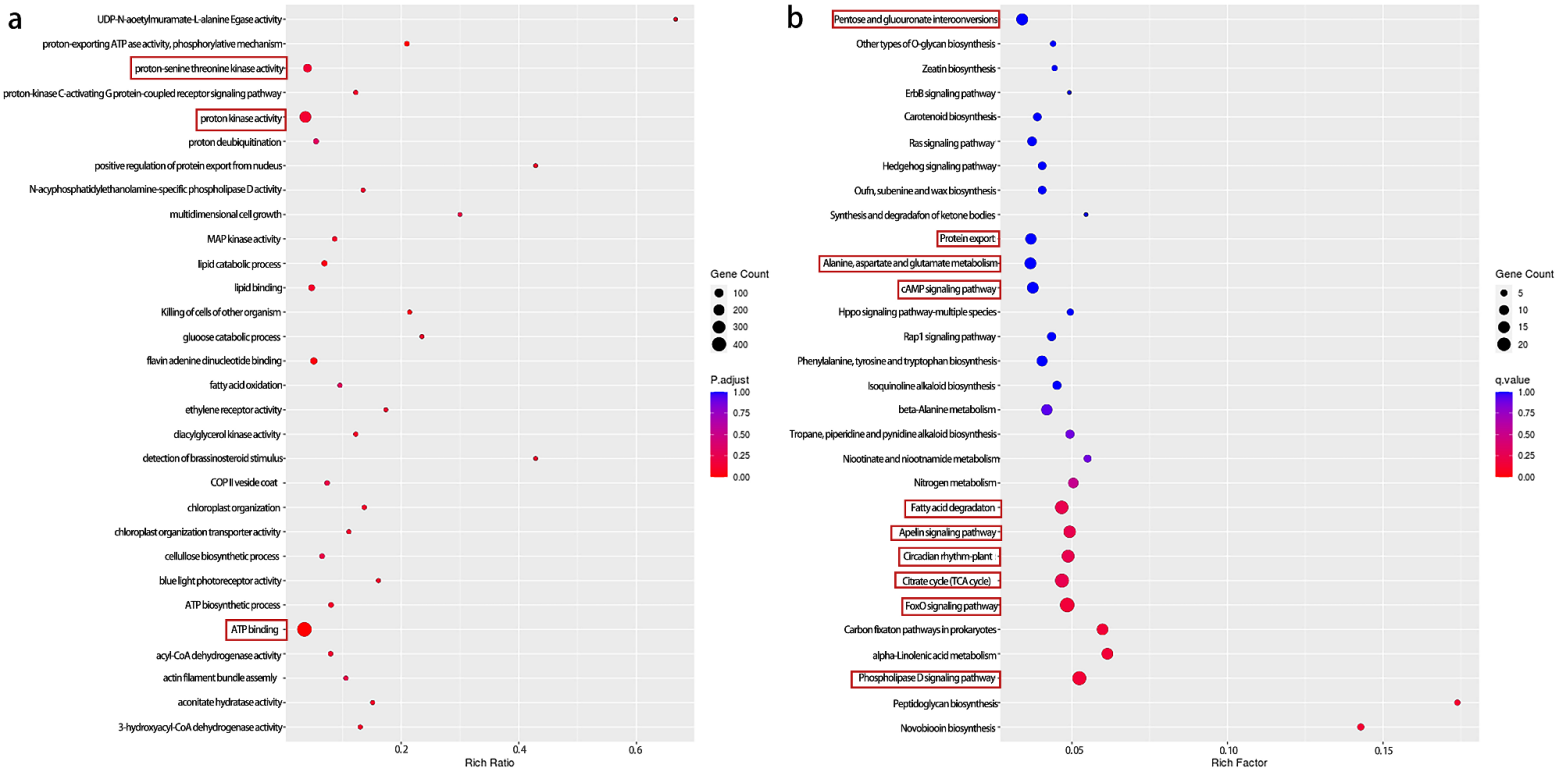
Functional enrichment analysis of DEGs. (**a**) GO enrichment analysis of DEGs. (**b**) KEGG Pathway enrichment of DEGs. The X-axis is the enrichment rate, and the formula for calculating the Enrich factor is GeneRatio/BgRatio. The Y-axis represents the GO/KEGG term, which belongs to a classification described as the Class legend information on the right. Each dot represents a GO/KEGG term; the larger the dot, the more differentially expressed the genes are

The DEGs with FDR less than 0.1 were selected for GO classification enrichment analysis and KEGG pathway enrichment analysis. GO enrichment analysis results revealed that 421 DEGs were enriched for ATP binding in the stamen petaloid libraries group (PP) when compared with the non-petaloid (NP) libraries group. Protein kinase activity and protein serine/threonine kinase activity were highly enriched, suggesting that the physiological activity of the stamen petal was more pronounced relative to the normal petal (Fig. 4a). KEGG pathway enrichment analysis of the DEGs revealed that they were enriched into more than 130 pathways. A bubble map showed the top 30 entries with the largest number of enriched genes (Fig. 4b), which included pentose and glucuronate interconversion, protein export, alanine, aspartate and glutamate metabolism, cAMP signaling pathway, fatty acid degradation, apelin signaling pathway, circadian rhythm-plant, citrate cycle (TCA cycle), foxO signaling pathway, and phospholipase D signaling pathway. This finding suggests that these biological processes were potentially key in stamen petaloid development.

### Screening of DEGs associated with stamen petaloid

**Fig. 5:**
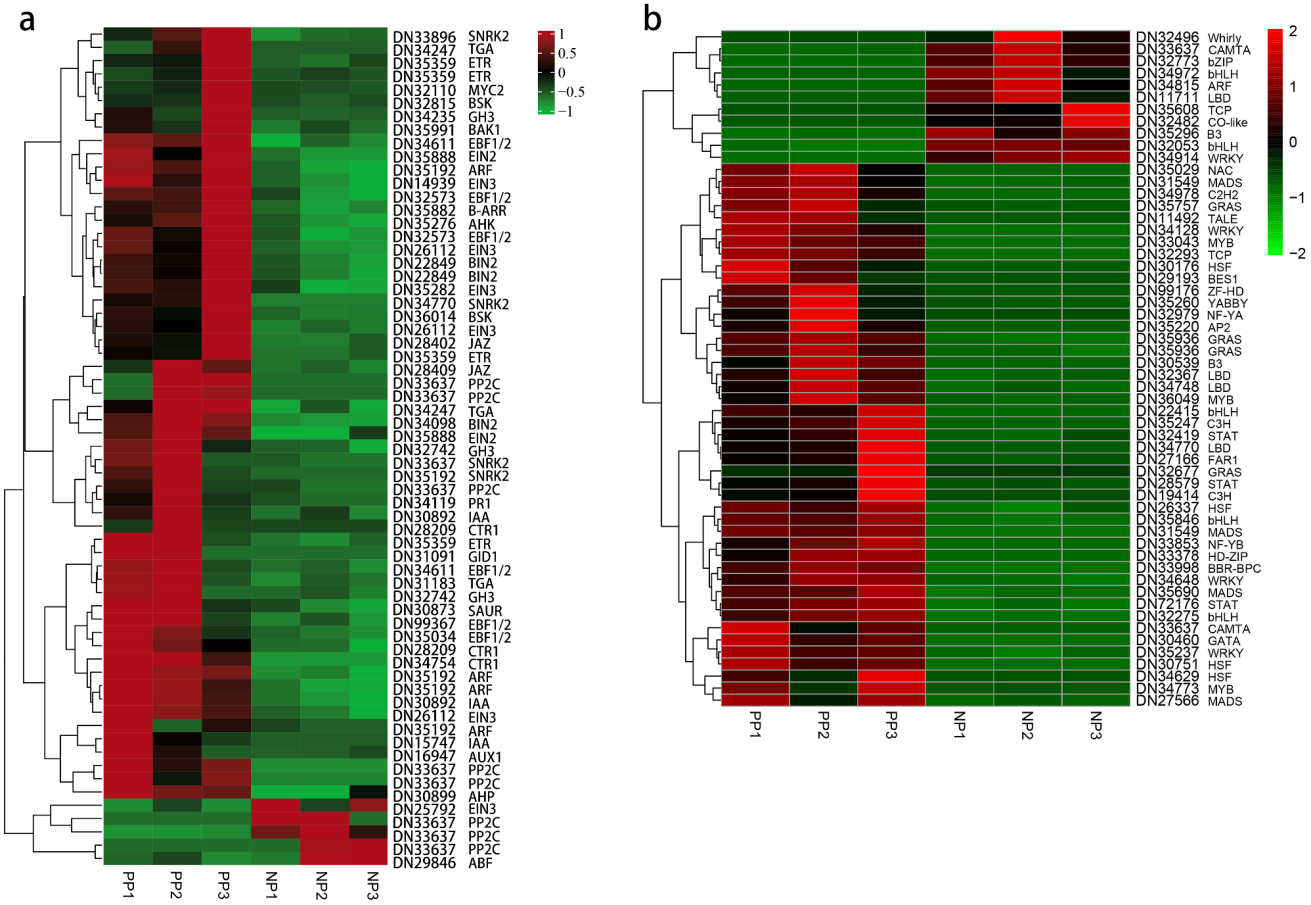
*A. rosea* stamen petaolid associated DEGs heat map clustering (**a**) Heat map of unigenes involved in plant hormone biosynthesis and signal transduction pathway. (**b**) Heat map of transcription factors associated with stamen petaloid. The bar represents the scale of the expression levels for each gene (log_10_RPKM) in PP vs. NP. The red bars represent up-regulated genes, the green bars represent down-regulated genes, and the black bars represent genes that do not differ significantly

**Fig. 6:**
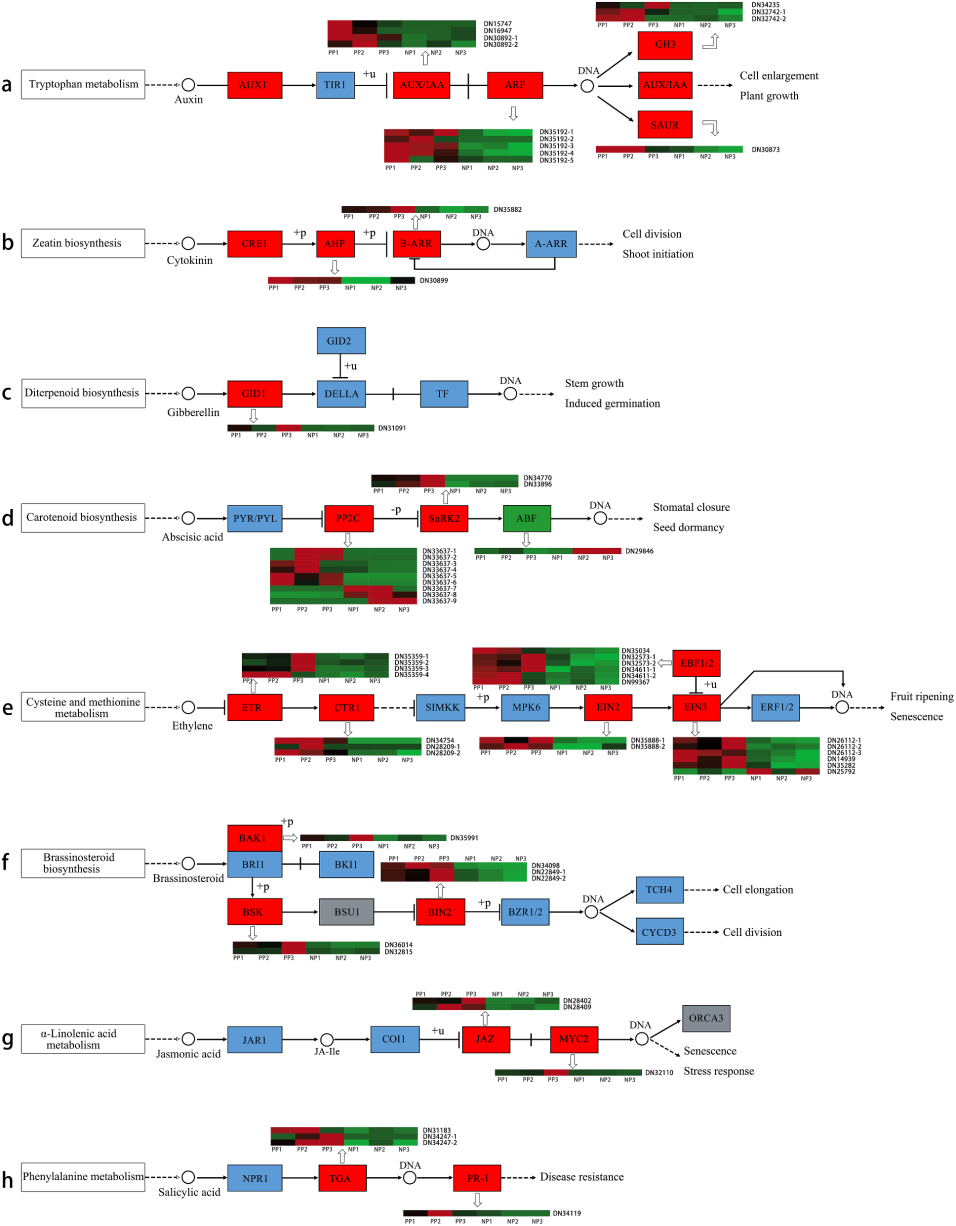
Analysis of plant hormone biosynthesis and signal transduction pathways. (**a**) Auxin signaling pathway. (**b**) Cytokinin signaling pathway. (**c**) Gibberellin signaling pathway. (**d**) Abscisic acid signaling pathway. (**e**) Ethylene signaling pathway. (**f**) Brassinosteroid signaling pathway. (**g**) Jasmonic acid signaling pathway. (**h**) Salicylic acid signaling pathway. The bar represents the scale of the expression levels for each gene (log_10_RPKM) in PP vs. NP. The red bars represent up-regulated genes, while the green bars represent down-regulated genes

Plant hormones play an important role in flower development by regulating the formation of anthocyanidin, the development of flower organs, and participating in nutrient metabolism and signal transduction [50–53]. Herein, transcriptome analysis revealed 63 genes involved in plant hormone biosynthesis and signal transduction pathway (ko04075) (Fig. 5a). The 63 genes belonged to different hormone regulation pathways, including auxin, cytokinin, gibberellin, abscisic acid, ethylene, brassinosteroid, jasmonic acid, and salicylic acid signaling pathways. *AUX/IAA* is a *ARF* repressor that undergoes proteasomal degradation upon auxin perception. *AUX/IAA*, small auxin-up RNA (*SAUR*), and the auxin-responsive *GH3* family protein (*GH3*) are the three major gene families involved in the early auxin response and regulate cell enlargement during plant growth and development [54]. There were 13 DEGs involved in auxin regulation pathways, including four *AUX/IAA* genes (DN15747, DN16947, DN30892-1, and DN30892-2), five *ARF* genes (DN35192-1, DN35192-2, DN35192-3, DN35192-4, and DN35192-5), three *GH3* genes (DN32742-1, DN32742-2, and DN34235) and one *SAUR* gene (DN30873) and were all up-regulated in PP compared to NP (Fig. 6a). In cytokinin (CTK) regulation pathways, genes downstream of *CRE1*, i.e., one *Arabidopsis* histidine-containing phosphotransfer (*AHP*) (DN30899) protein and one *B-ARR* gene (DN35882), were up-regulated in PP compared to NP, and they regulate cell division and shoot initiation (Fig. 6b). One GA insensitive dwarf1 (*GID1*) (DN31091) gene involved in gibberellin (GA) signal transduction in diterpenoid biosynthesis and in regulating stem growth in induced germination was up-regulated in PP compared to NP (Fig. 6c). In the abscisic acid (ABA) regulation pathways involved in carotenoid biosynthesis, six putative protein phosphatase genes (*PP2C*) (DN33637-1, DN33637-2, DN33637-3, DN33637-4, DN33637-5, and DN33637-6) and two sucrose nonfermenting 1 (*SNF1*)-related protein kinase 2 (*SnRK2*) genes (DN34770 and DN33896) were up-regulated, while three *PP2C* (DN33637-7, DN33637-8, and DN33637-9) and one *ABF* gene (DN29846) were down-regulated in PP compared to NP. These genes are ultimately involved in stomatal closure and seed dormancy (Fig. 6d). Additionally, there were 21 genes involved in ethylene signaling in cysteine and methionine metabolism and associated with fruit ripening and senescence. The genes included four ethylene-resistant (*ETR*) (DN35359-1, DN35359-2, DN35359-3, and DN35359-4), three constitutive triple response1 (*CTR1*) (DN34754, DN28209-1, and DN28209-2), two ethylene-insensitive2 (*EIN2*) (DN35888-1 and DN35888-2), six EIN3-binding F box protein 1/2 (*EBF1/2*) (DN35034, DN32573-1, DN32573-2, DN34611-1, DN34611-2, and DN99367). and six ethylene-insensitive3 (*EIN3*) genes (DN26112-1,DN26112-2, DN26112-3, DN14939, DN35282, and DN25792). All these genes were up-regulated in PP compared to NP except DN25792 (Fig. 6e). Six up-regulated genes in PP compared to NP were involved in brassinosteroid (BR) biosynthesis were identified. They included one BRI1-associated receptor kinase1 (*BAK1*) (DN35991), two brassinosteroid signaling kinase (*BSK*) (DN36014 and DN32815), and three brassinosteroid insensitive2 (*BIN2*) (DN34098, DN22849-1, and DN22849-2), and were potentially associated with cell elongation and division (Fig. 6f). Three up-regulated genes in PP compared to NP were involved in the jasmonic acid (JA) signaling pathway in α-linolenic acid metabolism were identified. They included two Jasmonate ZIM-domain proteins (*JAZ*) (DN28402 and DN28409) and one *MYC2* (DN32110) (Fig. 6g). In addition, four genes, including three TGACG motif binding factor (*TGA*) (DN31183, DN34247-1, and DN34247-2) and one pathogenesis-related protein-1 (*PR-1*) (DN34119), involved in salicylic acid signaling pathway in phenylalanine metabolism were up-regulated in PP compared to NP (Fig. 6h).

Transcription factors (TFs), such as members of the *MYB*, *bHLH*, and *WRKY* families, precisely control flower growth and development by combining hormone signals with environmental stimuli [55–57]. In this study, numerous TFs were determined to possibly play an important role in the process of stamen petaloid of *A. rosea*. In this study, 56 important TFs among the 3,212 DEGs were identified (Fig. 5b); 45 were up-regulated, while 11 were down-regulated and belonged to 28 TFs families. There were 5 up-regulated *MADS-box* TFs (DN35690, DN31549-2, DN31549-5, DN27566, and DN35220); 5 *bHLH* TFs (2 down-regulated *bHLH* TFs (DN32053 and DN34972) and 3 up-regulated *bHLH* TFs (DN35846, DN32275, and DN22415)); 4 up-regulated *GRAS* TFs (DN35936, DN32677, DN35757, and DN35936); 4 up-regulated *HSF* TFs (DN34629, DN30176, DN26337, and DN30751); 4 *LBD* TFs (1 down-regulated *LBD* TF (DN11711) and 3 up-regulated *LBD* TFs (DN34770, DN34748, and DN32367)); 3 up-regulated *MYB* TFs (DN33043, DN36049, and DN34773); 3 up-regulated *STAT* TFs (DN28579, DN32419, and DN72176); and 4 *WRKY* TFs (1 down-regulated *WRKY* TF (DN 34,914) and 3 up-regulated *WRKY* TFs (DN34128, DN34648, and DN35237)). In addition, other differentially expressed transcription factors included *ARF*, *B3*, *bZIP*, *C2H2*, *C3H*, *CO-like*, *FAR1*, *GATA*, *TCP*, *Whirly*, and *NAC*.

### Validation of RNA-seq data by qRT-PCR

**Fig. 7:**
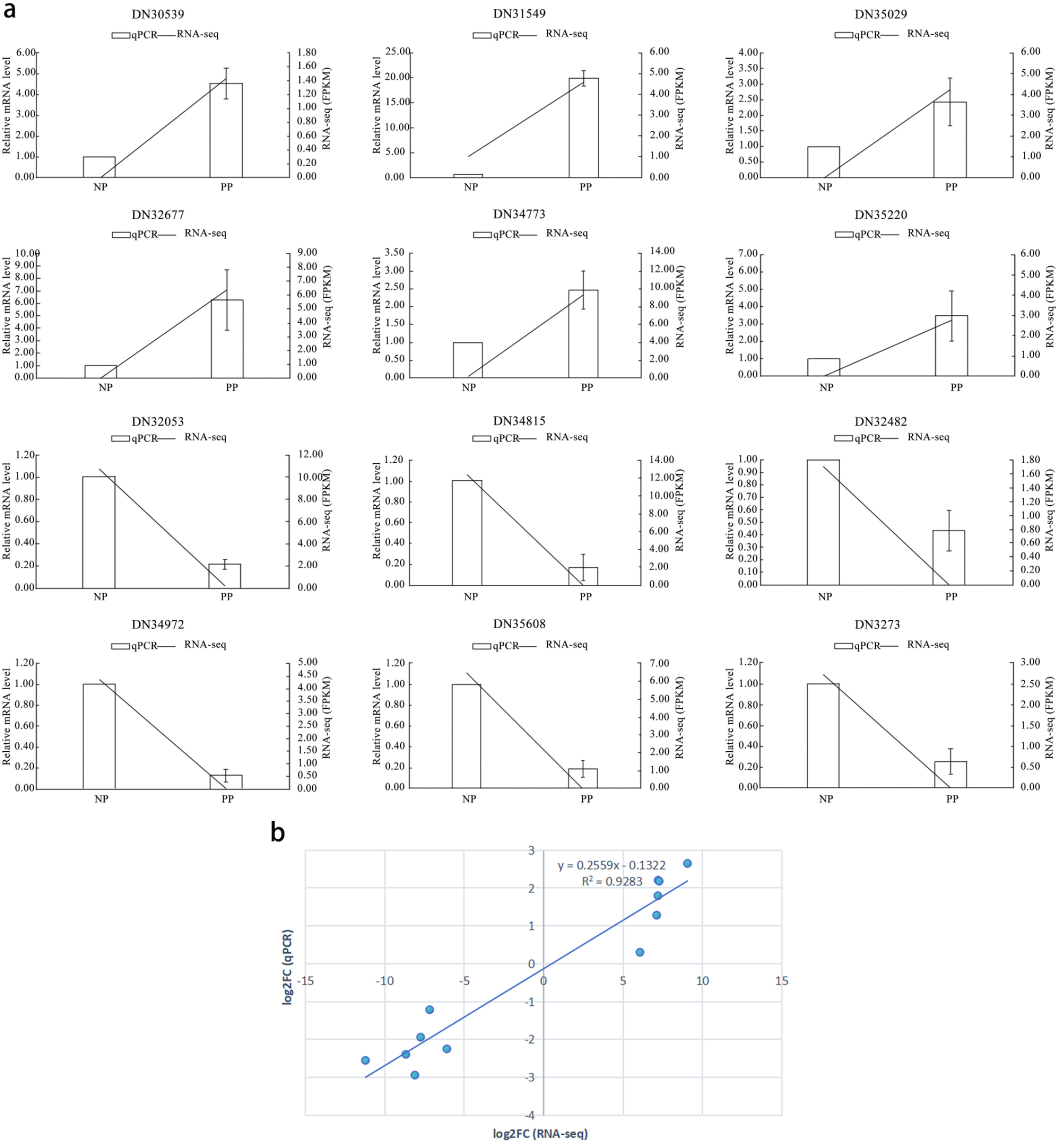
Validation of *A. rosea* stamen petaloid identified DEGs. (**a**) Relative expression levels of the twelve identified DEGs by qRT-PCR (bar chart, left Y-axes) and by FPKMs (lines, right Y-axes). 18s rRNA gene was used as reference for relative expression measurement in both qRT-PCR and RNAseq (FPKMs). Error bars indicate the standard deviation of three independent replicates (in qRT-PCR). (**b**) Fold-change value correlation analysis. RNA-seq fold change refers to the ratios of FPKM values of PP to NP, while qRT-PCR fold change is the relative quantity of PP normalized to the expression level of NP

Twelve DEGs were selected for qRT-PCR analysis to verify the reliability of RNA-seq data. Six DEGs, namely *AGL11* (DN31549), *AP2* (DN35220), *MYB* (DN34773), *NAC* (DN35029), *B3* (DN30539), and *GRAS* (DN32677), were up-regulated, while the other six, namely *bHLH* (DN32053), *bHLH* (DN34972), *ARF* (DN34815), *TCP* (DN35608), *bZIP* (DN32773), and *CO*-Like (DN32482) were down-regulated. Notably, the expression trend of the qRT-PCR results was consistent with the RNA-seq data (Fig. 7a). A linear regression analysis showed a 92.83% correlation between RNA-seq and qRT-PCR data (*R* = 0.9283) (Fig. 7b), indicating that the transcriptome data were reliable.

## Discussion

*A. rosea* is a common ornamental plant with bright flower color and rich flower type, that are now widely used around the world [43]. Among them, the *A. rosea* double-petal variety shows a graceful appearance that is being selected for the practical application in ornamental purposes. However, the regulation mechanism that turns stamen into stamen petaloid petals remains largely unclear. In this work, we identified the key genes related to stamen petaloid development and maturation in *A. rosea* by comparative transcriptomic analysis between the normal petal and stamen petaloid petal, laying an important foundation for further revealing the molecular mechanism of double-flower formation.

### Plant hormones regulate stamen petaloid

Plant hormones play an important role in the growth and development of flowers. several studies postulate that the homologous transformation of stamen into petal is closely associated with the synthesis, transport, and signal transduction of plant hormones [3, 58]. In this work, we identified 63 hormone-related genes as DEGs associated with stamen petaloid development in *A. rosea*. Previous studies have shown that auxin biosynthesis, transport, and signaling are essential for the development of the stamen. The stamen is the male reproductive structure of a flower, and its development consists (1) the early stage of stamen formation and morphogenesis and (2) the late stage of pollen grain maturation, filament elongation, and anther dehiscence [59]. The most critical enzymes in auxin synthesis belong to the *YUC* family of flavin monooxygenase [60]. Auxins are synthesized and transported to the top of the stamen primordium in a directional way to control stamen development [59]. *AUX/IAA* and *ARF* genes are not only among the genes regulated by auxin but also among the proteins involved in nuclear auxin signaling [61–63]. For instance, the auxin receptor transport inhibitor response 1 (*TIR1*) and auxin signaling F-box protein (*AFB*) perceive the auxin signal and recruit *AUX/IAA* for ubiquitination, thereby releasing the auxin response factor (*ARF*) to activate the auxin response in *A. thaliana* [64]. This pathway is essentially universal and functions in other plants, and mutations in each of these genes may affect stamen development. *ARFs* play an important role in the flower development process. In *A. thaliana*, *AtARF1* and *AtARF2* affect flower initiation, stamen development, and flower senescence [65]. The IAA level is significantly and positively correlated with the expression profile of *OfARF11a*, *12*, *13*, and *14a* in *Osmanthus fragrans* flower development [66]. A previous study has found that *AtARF* mutations in *A. thaliana* lead to changes in the number of stamens and petals [67]. Increased expression of *PgARF* may facilitate the development of stamens into petals in *Punica granatum* [68]. In *Rosa rugosa*, the auxin-regulatory gene *RhARF18* encodes a transcriptional repressor of the class-C gene *RhAG*, which regulates the stamen-petal transition in an auxin-dependent manner [69]. Increased *AtARF17* expression levels in *A. thaliana* altered the accumulation of auxin-inducible *GH3*-like, and these expression changes are associated with significant developmental defects, including the reduction of petal size and abnormal stamen formation [70].

In addition to auxin, several other classes of phytohormones are also closely related to stamen development. Jasmonic acidis detected by COI1, which recruits JAZ proteins for degradation and activates transcription factors essential for stamen development, and mutations in these JA signaling components lead to failure of filament elongation, delayed anther dehiscence, and inactive pollen [71, 72]. *GID1* detects gibberellin signals and recruits DELLA proteins, which activate downstream pathways through degradation of the 26 S proteasome to promote GA responses, a process of signaling that affects stamen development [73]. It was shown that mutations of *GID 1a*, *GID 1b*, and *GID 1c* in *Arabidopsis* may lead to failure of filament elongation and arrested anther development [64]. Gibberellin promotes stamen development in *A*. *thaliana* by promoting the expression of *MYB* TFs, which are controlled by jasmonic acid [74]. The gibberellin, abscisic acid, salicylic acid, and methyl jasmonate (MeJA) signaling pathways may interact with *cis*-acting elements in the promoter region of the *MADS-box* gene to regulate stamen and petal development [34, 75]. Studies have shown that ethylene is critical in determining plant reproductive organs. Treatment of wheat with exogenous ethylene resulted in the appearance of shorter stamens or their transformation into pistils [76]. Ethylene activates *EIN2* and *EIN3* transcription and inhibits anther development through the *EIN2-EIN3*/*EIL1* signaling pathway [77]. The brassinosteroid and gibberellin signaling pathways are directly or indirectly regulated by *SEP* genes, and *SEP* mutations result in abnormal phenotypes, such as deficient anthers and pollen, as well as free stamen filaments [78]. The *SKP1* gene plays an important role in plants. For instance, mutations in *ASK1*, a homolog of *SKP1* in *A. thaliana*, which synergistically regulates the expression of class-B genes in cooperation with *UFO* and *LEAFY*, cause developmental defects in floral organs [79, 80]. In *Dimocarpus longan*, the promoters of the *DlSKP1* family members contain lots of abscisic acid and MeJA response elements [81]. Herein, *SKP1*-like21 (|log2FC|>6) was significantly up-regulated in *A. rosea*, highlighting that it potentially plays a role in the development of the stamen petaloid of *A. rosea*.

### Involvement of transcription factors in stamen petaloid

In our study, 56 TFs related to floral organ development were found to be significantly differentially expressed between the two groups of samples and were mainly up-regulated in PP vs. NP. Gao et al. (2022) also screened some key TFs related to stamen petaloid organs by transcriptional analysis of red double-petal and single-petal flowers of *A. rosea* [43]. Some of the results of the present study are consistent with theirs. for example, both studies assayed TFs of the *MYB*, *bHLH*, *WRKY*, *NAC*, and *GATA* families, and most of them were up-regulated in petaloid petals. Accordingly, it is hypothesized that the overexpression of these TFs may be closely related to stamen petaloid in *A. rosea*. Wherein, *MYB* interacts with *bHLH* TFs to form a *bHLH-MYB* transcription complex that regulates stamen development [82]. NAC expression is induced by ethylene to affect petal growth and development and regulates the size of petal cells [83]. *WRKY* is closely related to stamen development, floral primordial differentiation, and abiotic stresses [84–86]. In addition, some other transcription factors were identified in this study, which were hypothesized to be possibly associated with the stamen-to-petal developmental change in *A. rosea*. For instance, *C2H2-ZFP* is involved in the induction of flowering, flower organ development, and transcriptional regulation of pollen and pistil development [87]. In *Brassica rapa*, *BrZFP244* and other genes are highly expressed during stamen development, while *BrZFP187* is highly expressed in the pistil [88]. *TCP* TFs have an important effect on flower development. *TCP2* affects plant leaf morphology and flowering time and mediates the JA signal transduction pathway [89]. *CYC*-like is involved in defining the complex inflorescence structure of the composite family [90].

The ABCE model has been shown to regulate floral organ development, but the conservation of this model varies greatly among different plant taxa [91]. It was shown that the expression level of the *AG*-like gene is associated with the degree of stamen petaloid. For instance, in *A. thaliana*, stamens are transformed into petals when the function of *AG* genes is lost [92]. In the same line, the expression level of class C gene *LelAG1* in the 3rd and 4th wheel flower organ development decreases with an increase in the stamen petaloid degree of multiple-petal *Lilium brownii* [93]. Excessive accumulation of *AP2* reduces the expression level of *AG*, resulting in the homologous conversion of stamen to petal [94]. *AG* mutations are associated with semi-multiple petal flower formation in *Dianthus caryophyllus* [95]. *DcaAG* genes might affect the petal number negatively and have a specific function in stamen and carpel development in *D. caryophyllus* [96]. These studies collectively suggest that the multiple petal flowers formed by the stamen petaloid or pistil petaloid are potentially associated with the *AG* genes. In this study, the two *AG*-like genes, namely DN31549-2 and DN31549-5 among the five DEGs in the *MADS-box* family, were up-regulated in PP. Notably, a similar phenomenon is also observed in *Paeonia suffruticosa* [97]. When previous researchers analyzed the transcripts of normal and petaloid petals of *P. suffruticosa*, they found that the expression of *AG* was significantly higher in petaloid petals than in normal petals [97]. Gao et al. (2022) also found that the expression level of *AG*-Like genes was higher in stamen petaloid organs than in normal petals in *A. rosea* [43]. In lily ‘Elodie’, the highest expression of *LelAG1* genes was also observed in stamens, followed by petaloid petals, while the lowest expression was observed in normal petals [92]. Therefore, we hypothesized that since *AG* is mainly expressed in stamens, its down-regulation compared to normal stamens may result in a stamen-to-petal developmental transition; however, it is still expressed at a higher level in stamen petaloid petals than in normal petals. Overexpression of class-B genes has been found to cause developmental defects in stamens and the development of petals in many plants, such as *Petrocosmea* (Gesneriaceae), *Prunus persica* [98, 99]. However, no significantly differentially expressed class-B genes were found in this study, in agreement with the studies of stamen petaloid development in red double-petal flower *A. rosea* and *N. nucifera* [29, 43]. Therefore, we hypothesize that class-B genes in *A. rosea* may have little to do with the emergence of the phenomenon of stamen petaloid development. In future studies, the role of class-C genes, as well as other key TFs in the regulation of stamen petaloid in *A. rosea*, merits deep exploration and verification.

## Conclusions

The formation of stamen petaloid petals is a very complex process regulated by an interaction of complex regulatory networks. In this study, 3,212 DEGs (2,620 DEGs were up-regulated, while 592 DEGs were down-regulated) involved in stamen petaloid petal formation were identified through non-parametric transcriptome sequencing analysis of pink flowers of *A. rosea*. Among the identified DEGs 63 genes were identified as involved in the plant hormone regulation pathway of flower organ growth and development and, 56 genes identified as key TFs associated with stamen petaloid. We hypothesized that in *A. rosea* the stamen petaloid development is mainly regulated by class-C genes (DN31549-2 and DN31549-3) in the ABCE model, with the involvement of some other TFs and phytohormone-related genes. The findings of this study provide a basis for further research on innovative flower shape breeding programs.

### Electronic supplementary material

Below is the link to the electronic supplementary material.

Supplementary Material 1

## Data Availability

The datasets generated during the current study are available in the NCBI Sequence Read Archive (SRA) with bioproject No. PRJNA1083646.
