# Supplementary material for: Transcriptomics analyses reveal the key genes involved in stamen petaloid formation in Alcea rosea L
Source: BMC Plant Biol. 2024 Jun 14;24:551. doi: 10.1186/s12870-024-05263-6 (PMC11177533; doi:10.1186/s12870-024-05263-6)

**Supplementary Materials 1** The genes and primers required used for qRT-PCR validation.

| Genes ID | Forward sequence (5’-3’ ) | Reverse sequence (5’-3’ ) |
| --- | --- | --- |
| 18S | CTGAGAAACGGCTACCACAT | ACCCAAGGTCCAACTACGAG |
| DN31549 | TTGATGGCACCCGGAGAGTCTAG | AAGGGGCATTTGGAACTGTTGGG |
| DN35220 | GGCGGTGTCCTCTCCAACATTG | CCGAGAAGTTCCGACAGGCAAAC |
| DN32053 | GTACCACTCGCAGTGCTGTCAG | CCCTCAGCGTGGATAGCTTGTTC |
| DN34972 | GGCTCTTCTTTCGGGCTGTTCTG | AACCACGCCGCTGTATAAACTCC |
| DN34773 | AGCTCCCACTGCCAGGTTCTC | CCTTGTACTGCTTCACCGACTCC |
| DN34815 | TCCTAATGCCTCGGCTCTCTCTC | CACCAGCCTCCAGCCAACAAC |
| DN35029 | GCAGCAGCCGATTCAGTCCAG | GATGTTCAGGCGTGGCATTTGC |
| DN35608 | TCAACGACTTCACGAGGAATGCC | TTCGCATCGTAAAGGGGCTTCG |
| DN30539 | TGGTCGCTCCTCTGGCAGTTAG | GACCAACGCACGTTTTCTTGCAG |
| DN32677 | TCGGGACAGACTCTTGAGGTTGG | CAGGAAGCGGGGAATGAATGGG |
| DN32773 | GGAGTTAGCGAGGCAGAAGTTGG | CACCACCACCTGGCACAATACC |
| DN32482 | GAGTCCAAGGCAAGTTCCGAAGG | AACCACGGCCTCTGCTTGAAAC |

Note:The gene number is automatically numbered by trinity software, for example, DN35192 represents a gene seat, followed by -1, -2, -3, which means that according to the sequence assembly it is presumed that this gene seat may be transcribed with 3 different transcripts, that is, the variable shear body.

**Supplementary Materials 2** Statistics of species distribution in the NR database.


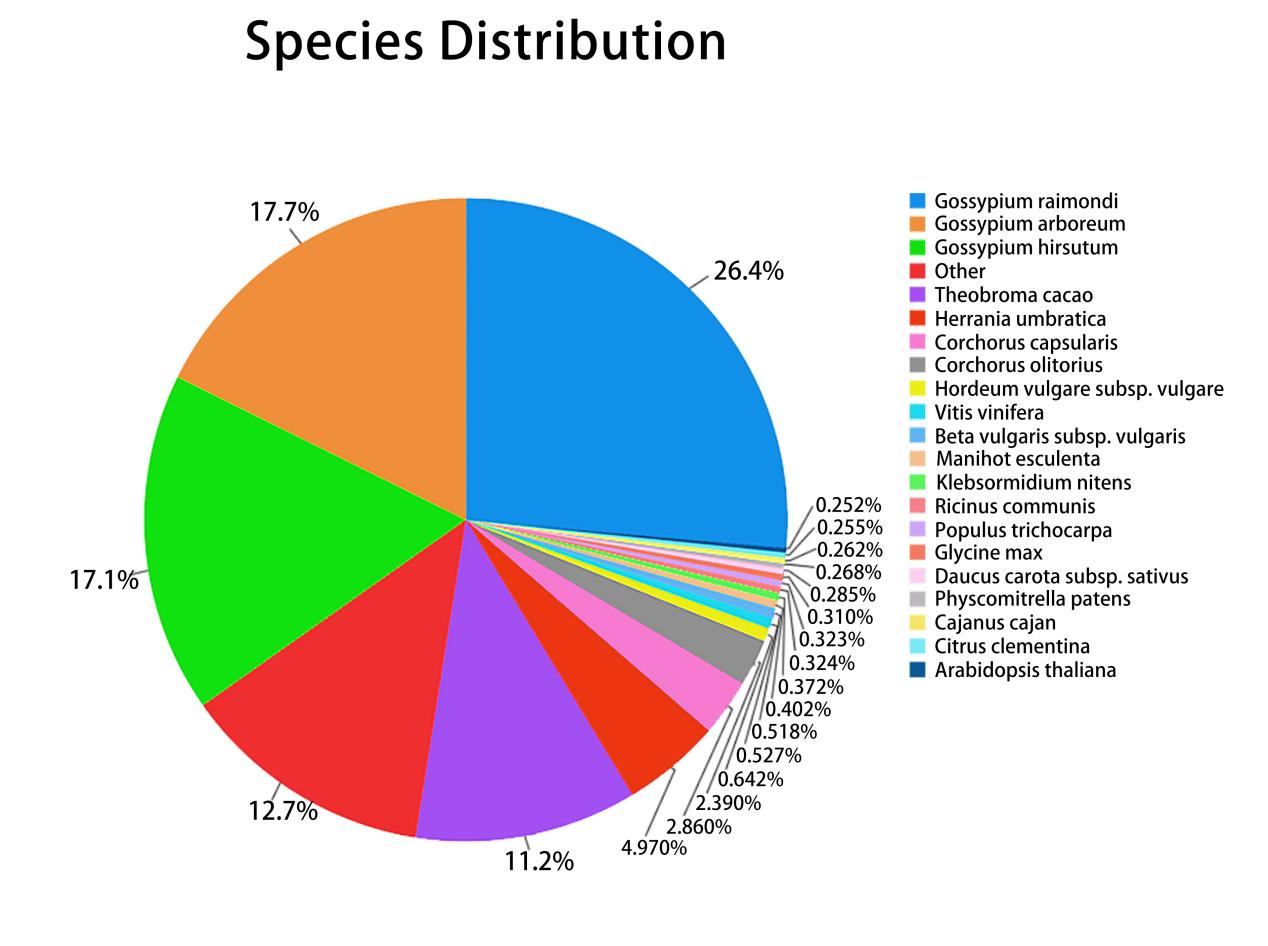

Supplement: Supplementary file 1 — Supplementary Material 1 [file 12870_2024_5263_MOESM1_ESM.docx]
